# Physiological, anatomical, and molecular responses of glanded and glandless cotton to chromium exposure

**DOI:** 10.3389/fpls.2025.1715493

**Published:** 2026-01-07

**Authors:** Samrana Samrana, Abid Ali, Sayed Hussain, Hamid Ali, Uzair Muhammad, Kasim Sakran Abass

**Affiliations:** 1 College of Agriculture and Biotechnology, Zhejiang University, Hangzhou, Zhejiang, China; 2 Department of Botany, University of Swabi, Swabi, Khyber Pakhtunkhwa, Pakistan; 3 Department of Horticulture, Faculty of Chemical and Life Sciences, Abdul Wali Khan University Mardan, Mardan, Khyber Pakhtunkhwa, Pakistan; 4 Department of Bioscience, Comsats University, Islamabad, Pakistan; 5 College of Veterinary Medicine Department of Physiology, Biochemistry and Pharmacology, University of Kirkuk, Kirkuk, Iraq

**Keywords:** antioxidant activities, chromium (Cr), cotton, gene expression, gossypol, leaves ultrastructure

## Abstract

Cotton seeds are a major source of high-quality proteins and edible oil, but their utilization is limited due to gossypol toxicity to humans and animals. For better use of cotton as food and feed, several glandless cotton cultivars have been developed, which are susceptible to various biotic stresses. However, their resistance to abiotic stresses, i.e., heavy metals, has rarely been studied. In the current study, the effect of different doses of chromium (Cr)—0, 10, 50, and 100 μM—on the physiological, anatomical, and molecular aspects of cotton was investigated. Genotypic variation exists in the response of cotton to Cr stress. Current results revealed that Cr caused inhibition in leaf biomass and ultrastructure damage, showing large intercellular spaces, thick cell walls, distorted nucleus and chloroplast, and a ruptured nuclear membrane. Cr affected the biochemical system and resulted in the reduction of net photosynthesis rate by 69.7%, intercellular CO_2_ by 84%, and stomatal conductance by 68%. Thiobarbituric acid-reactive substances and H_2_O_2_ increased with increasing Cr concentration, with relatively higher levels in glanded cotton. The expression of genes (*GhSOD*, *GhPOD*, *GhAPX*, *GhCAT*) encoding antioxidant enzymes in the leaves was increased, helping to maintain the activity of antioxidant compounds. According to the results, it was observed that the antioxidant activity of GhSOD, upregulated 2.5-fold at 100 μM of Cr, plays a key role in mitigating Cr-induced oxidative stress. This study revealed the response mechanism of Cr stress in glanded and glandless cotton that might perform different mechanisms to cope with Cr toxicity.

## 1 Introduction

Gossypol is mainly stored in the pigment gland of cotton plants, especially in their seeds, playing an important role in plant protection against insects and pests. However, it is toxic to humans and some animals (Ghorbani et al., 2025), which limits the widespread commercial use of cotton seeds. Since the 1960s, many studies have been performed to remove gossypol, and various glandless genotypes with low gossypol content in seeds and whole plants have been developed (Zhang et al., 2014). The commercial utilization of glandless cotton is limited due to its susceptibility to insects and pests. However, the response of glandless cotton to heavy metals is hardly found in the literature.

Contamination by heavy metals such as chromium (Cr), lead (Pb), cadmium (Cd), mercury (Hg), and arsenic (As) is among the most serious environmental problems, which not only pollutes the quality of soil, water, and air but also impacts the development of microorganisms, animals, and plants (Houda et al., 2016; Gupta et al., 2013; Boyd, 2010; Rascio and Navari-Izzo, 2011; Sánchez, 2008; McLaughlin et al., 2000). Cr is a hazardous heavy metal, which is present naturally in the environment (Sharma et al., 2023; Wu et al., 2025). It is released through anthropogenic activities, such as from paint production and wood preservation industries. Cr is found in different oxidation states (0–VI) (Khalofah, 2024). Among them, Cr (III) and Cr (VI) are the most common states, and Cr (VI) is considered more toxic to living organisms than Cr (III) (Kharbech et al., 2017). In plants, Cr disturbs various metabolic systems by affecting nutrient uptake and the metabolism of carbohydrates, and it also inhibits photosynthesis, root growth, plant biomass, and leaf chlorosis, which would lead to plant damage and cell death by creating lipid peroxidation and protein oxidation (Scoccianti et al., 2006; Sharma et al., 2003; Hayat et al., 2012). From the research background, it is clear that Cr (VI) causes phytotoxic injuries in various plant species, including pea (Pandey et al., 2009), wheat (Shaikh et al., 2013), tomato (Li et al., 2016), and maize (Mahajan et al., 2013).

Plant cells contact with Cr (VI) through the root meristem cells by consuming energy (Hayat et al., 2012; Shanker et al., 2005). In plants, it has been stated that the transportation rate of Cr (VI) is higher than its reduction as compared to Cr (III). Therefore, plants activate the mechanism of detoxification (Kaszycki et al., 2005; Sonali et al., 2010). Cr-induced damage in plants causes a shortage of essential nutrients and water in aboveground plant parts and finally disturbs the normal uptake of nutrients by creating oxidative damage. The uptake of a high amount of Cr in the roots is further translocated into the shoots, causing oxidative damage in the photosynthetic system as well as in the mitochondria, and finally results in stunted plant growth (Mathur et al., 2016). Under Cr treatments, plants induce ROS that leads to the production of harmful biomolecules, i.e., lipids and proteins, which would disturb carbohydrate metabolism and mitochondrial respiration (Gill and Tuteja, 2010; Gill et al., 2015). Plant photosynthesis under Cr stress is affected, including CO_2_ fixation, electron transport, enzymatic activities, and photophosphorylation (Mathur et al., 2016). When plants are exposed to toxic environmental conditions, they increase the production of ROS (H_2_O_2,_ O_2_^−^), TBARS, etc., and this response to Cr may serve as the first biochemical signal (Jasinski et al., 2008; Yadav, 2010; Grover et al., 2010; Lushchak, 2011; Opdenakker et al., 2012). Due to the production of ROS, an imbalance in their elimination occurs that creates oxidative stress (Morina et al., 2010). In order to overcome these challenges and to create a balanced environment, plants activate the defense system of antioxidant enzymes. Superoxide dismutase (SOD) acts as the first scavenging enzyme by converting O_2_^−^ to H_2_O_2_; furthermore, CAT, POD, and APX convert H_2_O_2_ to O_2_ and H_2_O (Anjum et al., 2015a; Kapoor et al., 2019).

The present study was conducted to investigate the differential mechanism of Cr tolerance in glanded and glandless cotton nearly isogenic lines (NILs) through photosynthetic, physiological, and molecular aspects.

## 2 Materials and methods

### 2.1 Plant materials and growth conditions

Glanded (ZMS-12, ZMS-16, ZMS-17, and Coker-312) and glandless cotton NILs (ZMS-12W, ZMS-16w, ZMS-17w, and Coker-312w) were used as plant materials in the experiment conducted at the College of Agriculture and Biotechnology, Zhejiang University P.R. China. The seeds of glandless NILs were obtained from the Cotton Research Centre, Chinese Academy of Agricultural Sciences, China. Seed sterilization and growth conditions were according to the protocol of Samrana et al. (2020) with slight modifications. The seeds were soaked in double-distilled water at 28°C for 24 h. For germination, the seeds were sown in moist peat mass in a controlled environment under a 16/8-h light/dark photoperiod with white fluorescent light at 50 μmol^−2^ s^−1^, temperature of 28 °C ± 2 °C, and 60% relative humidity for 7 days. Subsequently, uniform seedlings were transplanted into Hoagland solution according to Samrana et al. (2020); Daud et al. (2014), and Muhammad et al. (2024) (pH 5.6–5.7) and grown under a 14/10-h light/dark cycle at 30°С ± 2°С. After 14 days, the seedlings were exposed to 0, 10, 50, and 100 μM of Cr^6+^ (K_2_Cr_2_O_7_) for 7 days, with each treatment replicated three times. The nutrient solution was changed every 3 days and aerated with an air pump. Morphological parameters like fresh dry biomass were measured from 10 plants using an electronic balance, while plant height was manually calculated using an inch tape.

### 2.2 Gas exchange

Net photosynthetic rate (P_n_), stomatal conductance (G_s_), intercellular CO_2_ concentration (C_i_), and transpiration rate (E) in the third-leaf stage were measured using an open-flow infrared gas analyzer (LI-COR 6400) (LI-COR, Lincoln, NE, USA) and an LED light source. The light intensity, leaf temperature, and CO_2_ concentration inside the leaf chamber were kept constant as follows: 400 ± 2 μmol m^−2^ s^−1^, 25 °C ± 0.3 °C, and 400 ± 5 μmol CO_2_ mol^−1^, respectively.

### 2.3 Determination of Cr (IV) and micro- and macronutrients in the seedlings

Three plants from each pot were selected and washed with tap water first and then with distilled water. Roots were immersed in 20 mM of EDTA-Na_2_ for 15 min to remove the adhering metals and then washed three times with ddH_2_O. Roots placed in brown paper bags were dried in an oven at 80°С for 48 h, and 0.2 g of the dried sample was digested in a mixture of a solution of HNO_3_ and HCLO_4_ (5:1, v/v). The digested sample was diluted with 2% HNO_3_ to a final volume of 25 mL and filtered. The filtrate was used for the analysis of Cr and other microelements (Fe, Zn, Mn, and Na) and macroelements (Ca, S, K, P, and Mg) using an atomic absorption spectrometer (iCAT-6000-6300, Thermo Scientific, USA).

### 2.4 Chlorophyll contents

For the investigation of photosynthetic pigments, chlorophyll a (Chl *a*), chlorophyll b (Chl *b*), and carotenoids were measured. Fresh leaf samples (25 mg) were combined with magnesium oxide (25 mg) for the prevention of pheophytin, after which 5 mL of 100% methanol was added and homogenized in a dark environment for at least 2 h and then kept overnight in the dark at 25°C. Afterward, the samples were centrifuged at 4,000 rpm for 15 min at 25°C. For chlorophyll measurements, 1 mL of the supernatant was used in a cuvette, and the values were recorded at 470, 653, and 666 nm (Spectrum SP-752, Shanghai, China). Chl *a*, Chl *b*, and carotenoids were measured following the proposed method of Lichtenthaler and Wellburn (1983).

### 2.5 Total soluble proteins

Total soluble protein (TSP) concentration in the leaves was determined according to a colorimetric method utilizing a protein assay dye (Coomassie Brilliant Blue G-250). The absorbance was determined at 595 nm (Bradford, 1976).

### 2.6 Determination of lipid peroxidation and H_2_O_2_

Lipid peroxidation was measured according to Chappell and Cohn (2011). Fresh leaf samples (0.5 g) were extracted according to Samrana et al. (2020) with some modifications; the extraction of the fresh leaf samples was done in 10 mL of 0.25% (w/v) TBA prepared in 10% (v/v) trichloroacetic acid (TCA). The extract was heated in a water bath at 95°C for 30 min, cooled on ice, and centrifuged at 10,000 rpm for 10 min. The absorbance of the supernatant was measured at 532 and 600 nm, and hydrogen peroxide (H_2_O_2_) concentration was measured according to Velikova et al. (2000) using a Spectrum SP-752 spectrophotometer (Shanghai, China).

### 2.7 Antioxidant enzyme activity

Fresh leaf samples (0.5 g) were homogenized in 8 mL of 50 mM potassium phosphate buffer (pH 7.0 containing 1 mM of EDTA Na_2_ and 0.5% PVP w/v) under cold conditions. The homogenate was centrifuged at 12,000 rpm for 20 min at 4°С. The supernatants were collected in separate tubes and stored at −80°С. For the measurement of SOD, the method of Giannopolitis and Ries (1977) was used. POD EC 1.11.1.7 activity was measured according to Zhang (1992). CAT activity was examined using the method of Aebi and Aebi (1984). APX activity was determined by following the protocol of Nakano and Asada (1981).

### 2.8 Ultrastructure analysis

Fresh leaf samples from both glanded and glandless cotton NILs under control and stressed plants were collected in 2.5% glutaraldehyde in 0.1 M of PBS (sodium phosphate buffer) at pH 7.4. The samples were then washed with 0.1 M of PBS and fixed with osmium oxide for 1 h. The samples were washed again with the same PBS four times with 15 min intervals. Then, a series of graded ethanol (50%, 70%, 80%, 90%, and 100%) was used for dehydration at 15 min intervals. For further dehydration, absolute acetone was used for 15–20 min. The samples were embedded overnight in Spurr resin and heated for 9 h at 70°C. The sections (80 nm) were prepared, mounted on copper grids, and examined through an electron microscope (JEOL TEM-1230X) at the Electron Microscopic Centre, Zhejiang University, Hangzhou, China.

### 2.9 RNA extraction and qPCR

For RNA extraction, approximately 50 mg of fresh leaf sample was ground in liquid nitrogen using a mortar and pestle and then processed with RNAiso reagent (TaKaRa, Nojihigashi, Japan). RNA concentration was determined using the Thermo 200 Bioanalyzer NanoDrop (Thermo Scientific, Waltham, MA, USA, http://www.thermo.com). For reverse transcription (1 µg in 20 µL), extracted RNA was used, applying the ReverTra Ace qPCR master mix with a gDNA remover kit (Toyobo, Osaka, Japan) and diluting in 200 µL of water. PCR mixture (20 µL) and 2.5 µL of single cDNA were used for qPCR analysis, along with 10 µL of 2 *×* FastStart Universal SYBR Green Master (Toyobo, Osaka, Japan) and 0.25 µM of forward and reverse primers. Three biological replicates were used for gene expression, and CT values were obtained from the RT-qPCR system—StepOne v.2.1 software (Applied Biosystems, United State of America). ΔCT values were calculated between gene CT and GhUBQ7 CT values, with GhUBQ7 being used as the internal standard. The primers used for the expression are presented in **Table 1**.

**Table 1:** Primers used for the expression of antioxidant gene expression.

Gene Name	Primer	Sequence 5′ to 3′
*GhUBQ7*	Forward	AGAGGTCGAGTCTTCGGACA
	Reverse	GCTTGATCTTCTTGGGCTTG
*GhSOD*	Forward	CCACTTAGCGGAGAGCA
	Reverse	GCACCTTCATTTCCCTGTC
GhCAT	Forward	CAGCCATGCCACTCAGGATCTC
	Reverse	TGCAAGCTGTTCATTTTCGGCG
*GhAPX*	Forward	CTGATGAGGATGCTTTCTTTGC
	Reverse	AACCTAAAACGTCTGTGTAGGATTG
*GhPOD*	Forward	TTCAATTCGGATTTTGTGGC
	Reverse	TGCAGAAATTAGTTCACCCT

### 2.10 Statistical analysis

Analysis of variance (ANOVA) with the least significant difference (LSD) as a *post hoc* test at a 95% confidence interval was used to assess the significance of differences among various data sets using Statistix 9.0. Means and standard deviations of three replicates of each set were calculated using the same software.

## 3 Results

### 3.1 Effect of Cr on fresh and dry biomass and plant height

The effect of Cr on the fresh biomass of leaves and stems was investigated in both glanded and glandless cotton NILs. There was no difference observed in the stem fresh weight of glanded NILs between the control and 10 μM of Cr treatment, whereas it was significantly reduced by increasing Cr concentrations in glandless NILs. Glanded ZMS-12 and ZMS-17 showed a higher relative decrease at 50 μM (43% and 64%) and 100 μM (60% and 73%) as compared to their glandless NILs at 50 μM (37% and 47%) and 100 μM (52% and 64%), respectively. Meanwhile, it was the opposite in Coker-312, where the glandless NILs showed the highest decrease at 50 μM (56%) and 100 μM (70%) than their glanded NIL, decreasing by 5% and 46%, respectively. From the average data, the stem fresh weight of glandless NILs was lower at 10 μM but higher at 50 and 100 μM than their glanded NILs.

Under controlled conditions, the leaf biomass was significantly higher compared to the treatments, which was in the range of 2.65~3.38 g in glanded NILs and 2.90~4.22 g in glandless NILs. The highest reduction in fresh leaf biomass was observed at 100 μM in ZMS-16w and its glanded NILs, only 87% and 84% of the control, respectively. In comparison, the fresh weight of the leaves in glandless NILs was higher in control and 10 μM as compared to their glanded NILs (**Table 2**). From the above results, it is clear that glandless cotton showed less effect on fresh biomass under Cr stress as compared to glanded cotton.

**Table 2:** Effect of Cr on leaf and stem biomass and plant height of four pairs of glanded and glandless cotton NILs.

Parameters	Cr levels	NIL-1		NIL-2		NIL-3		NIL-4		Average	
		ZMS-17 (Glanded)	ZMS-17w (Glandless)	ZMS-16 (Glanded)	ZMS-16w (Glandless)	ZMS-12 (Glanded)	ZMS-12w (Glandless)	Coker-312 (Glanded)	Coker-312w (Glandless)	Glanded	Glandless
Leaf fresh weight (g)	Control	2.72 ± 0.51^ab^	3.39 ± 0.60^a^	3.18 ± 0.50^ab^	3.72 ± 0.44^a^	3.38 ± 0.42^b^	4.22 ± 0.59^a^	2.65 ± 0.15^b^	3.17 ± 0.20^a^	2.93 ± 0.33^a^	3.49 ± 0.58^a^
	10 µM	2.54 ± 0.24^b^	2.15 ± 0.30^c^	2.49 ± 0.32^c^	3.04 ± 0.62^bc^	2.33 ± 0.48^c^	3.13 ± 0.36^b^	2.63 ± 0.03^b^	2.40 ± 0.26^b^	2.44 ± 0.18^b^	2.44 ± 0.58^b^
	50 µM	1.27 ± 0.21^cd^	1.26 ± 0.15^de^	1.22 ± 0.13^d^	1.34 ± 0.30^d^	1.73 ± 0.17^cd^	2.13 ± 0.37^c^	0.93 ± 0.15^d^	1.41 ± 0.10^c^	1.21 ± 0.38^c^	1.42 ± 0.51^c^
	100 µM	0.89 ± 0.11^e^	0.86 ± 0.19^e^	0.45 ± 0.07^e^	0.51 ± 0.05^e^	1.00 ± 0.21^e^	1.10 ± 0.22^de^	0.83 ± 0.08^d^	0.56 ± 0.12^d^	0.80 ± 0.22^c^	0.76 ± 0.28^c^
Stem fresh weight (g)	Control	1.36 ± 0.06^a^	1.39 ± 0.21^a^	1.44 ± 0.25^a^	1.39 ± 0.33^a^	1.28 ± 0.13^a^	1.29 ± 0.50^a^	0.88 ± 0.05^b^	1.24 ± 0.07^a^	1.24 ± 0.24^a^	1.31 ± 0.10^a^
	10 µM	1.09 ± 0.09^a^	1.07 ± 0.10^b^	1.27 ± 0.18^a^	0.18 ± 0.19^a^	1.02 ± 0.11^ab^	1.06 ± 0.05^ab^	0.88 ± 0.03^b^	0.96 ± 0.05^b^	1.03 ± 0.10^a^	0.95 ± 0.09^b^
	50 µM	0.49 ± 0.11^c^	0.62 ± 0.14^c^	0.60 ± 0.02^b^	0.60 ± 0.09^b^	0.73 ± 0.06^bc^	0.82 ± 0.10^bc^	0.48 ± 0.02^c^	0.55 ± 0.08^c^	0.56 ± 0.11^b^	0.65 ± 0.12^c^
	100 µM	0.37 ± 0.06^d^	0.48 ± 0.06^cd^	0.46 ± 0.16^b^	0.45 ± 0.09^b^	0.51 ± 0.05^c^	0.62 ± 0.11^c^	0.47 ± 0.04^c^	0.37 ± 0.05^d^	0.46 ± 0.05^b^	0.47 ± 0.10^d^
Leaf dry weight (g)	Control	0.25 ± 0.04^ab^	0.32 ± 0.06^a^	0.38 ± 0.06^a^	0.42 ± 0.06^a^	0.33 ± 0.01^ab^	0.36 ± 0.05^a^	0.28 ± 0.00^b^	0.35 ± 0.04^a^	0.31 ± 0.05^a^	0.37 ± 0.04^a^
	10 µM	0.28 ± 0.03^a^	0.12 ± 0.03^bc^	0.30 ± 0.05^b^	0.37 ± 0.03^ab^	0.24 ± 0.04^c^	0.29 ± 0.05^bc^	0.28 ± 0.02^b^	0.25 ± 0.01^b^	0.28 ± 0.02^a^	0.29 ± 0.07^b^
	50 µM	0.17 ± 0.03^cd^	0.13 ± 0.02^de^	0.14 ± 0.01^cd^	0.20 ± 0.05^c^	0.24 ± 0.02^c^	0.24 ± 0.03^c^	0.12 ± 0.01^de^	0.18 ± 0.03^c^	0.17 ± 0.04^b^	0.19 ± 0.04^c^
	100 µM	0.09 ± 0.00^e^	0.08 ± 0.04^e^	0.05 ± 0.01^d^	0.06 ± 0.00^d^	0.12 ± 0.02^d^	0.12 ± 0.02^d^	0.16 ± 0.03^cd^	0.08 ± 0.00^e^	0.11 ± 0.04^b^	0.09 ± 0.02^d^
Stem dry weight (g)	Control	0.09 ± 0.01^abc^	0.10 ± 0.01^ab^	0.13 ± 0.03^bc^	0.14 ± 0.03^b^	0.10 ± 0.00^bc^	0.11 ± 0.01^bc^	0.08 ± 0.00^bcd^	0.13 ± 0.02^a^	0.11 ± 0.02^a^	0.12 ± 0.01^a^
	10 µM	0.10 ± 0.01^a^	0.08 ± 0.00^bcd^	0.13 ± 0.03^bc^	0.22 ± 0.04^a^	0.11 ± 0.00^b^	0.14 ± 0.02^a^	0.09 ± 0.02^bc^	0.10 ± 0.00^b^	0.11 ± 0.01^ab^	0.14 ± 0.06^a^
	50 µM	0.08 ± 0.01^cde^	0.06 ± 0.01^def^	0.08 ± 0.01^cd^	0.09 ± 0.03^bcd^	0.09 ± 0.00^bcd^	0.09 ± 0.01^bcd^	0.07 ± 0.01^bcd^	0.09 ± 0.01^bc^	0.09 ± 0.00^bc^	0.09 ± 0.01^ab^
	100 µM	0.04 ± 0.06^f^	0.05 ± 0.00^ef^	0.05 ± 0.02^d^	0.05 ± 0.00^d^	0.08 ± 0.01^d^	0.08 ± 0.01^cd^	0.07 ± 0.01^cd^	0.06 ± 0.01^d^	0.07 ± 0.01^c^	0.07 ± 0.01^b^
Plant height (cm)	Control	22.0 ± 0.48^a^	20.2 ± 1.52^ab^	22.8 ± 1.44^a^	20.8 ± 1.93^b^	20.6 ± 0.84^a^	21.1 ± 0.85^a^	18.1 ± 0.84^bc^	21.3 ± 0.32^a^	20.9 ± 2.05^a^	20.9 ± 0.49^a^
	10 µM	21.5 ± 0.77^ab^	19.9 ± 1.51^b^	22.1 ± 0.86^ab^	20.1 ± 0.62^b^	21.2 ± 0.85^a^	20.6 ± 0.35^a^	18.5 ± 0.50^b^	21.1 ± 0.35^a^	20.9 ± 1.57^a^	20.4 ± 0.53^a^
	50 µM	16.8 ± 1.88^c^	14.4 ± 0.61^d^	16.9 ± 0.52^c^	15.9 ± 0.83^c^	17.8 ± 0.86^b^	17.2 ± 1.80^b^	16.2 ± 0.81^de^	17.1 ± 1.00^cd^	16.9 ± 0.68^b^	16.2 ± 1.33^b^
	100 µM	12.1 ± 1.58^e^	12.9 ± 0.10^de^	13.9 ± 1.35^d^	13.5 ± 0.74^d^	15.40 ± 0.86^c^	15.0 ± 0.95^c^	15.1 ± 0.48^ef^	14.0 ± 0.90^f^	14.1 ± 1.49^c^	13.9 ± 0.88^c^

Data are the mean of three replications (mean ± SD). Different letters show significant differences at *P* < 0.05 for each pair of NILs at various Cr treatments.

The stem dry biomass of the control was 0.11~0.14 g/plant in glandless NILs, while it was 0.08~0.11 g/plant in the glanded NILs. The highest inhibition under Cr treatment was observed in ZMS-16 at 100 μM. Average data showed that stem dry biomass was reduced by 0.11 g/plant in glanded cotton, while in their glandless NILs, it was reduced by 0.14 g/plant at 10 μM. Stem dry biomass was reduced in glanded NILs as compared to glandless at 10 μM, while no changes were recorded at higher doses among glanded and glandless cotton. Furthermore, we also investigated the dry biomass of leaves in both glanded and glandless NILs, which showed a significant difference in the control and Cr treatments. The dry biomass values of leaves in glanded and their glandless NILs under control were 0.25~0.38 and 0.32~0.42 g/plant, respectively. The highest reduction (85%) in leaf dry biomass was observed in ZMS-16 NILs. The highest reduction was observed at 50 and 100 μM in glanded and glandless NILs, respectively.

Plant height of both glanded and glandless NILs was observed in the control and Cr-treated plants, which decreased with increasing concentrations of Cr. For the untreated glanded and their glandless NILs, 18.1~22.7 and 20.2~21.3 cm plant height was recorded, respectively. The highest reduction in plant height was recorded in ZMS-17 at 100 µM. Overall, plant height was decreased more significantly in glandless cotton than in glanded NILs (**Table 2**).

Photosynthesis is an important source of plant biomass yield. Under different concentrations of Cr (0, 10, 50, and 100 μM), gas exchange in terms of P_n_, G_s_, C_i_, and E in both glanded and their glandless NILs was investigated.

The P_n_ values in untreated plants of ZMS-16 and ZMS-17 showed no difference with their glandless NILs, while in Coker-312w and ZMS-12w, the P_n_ values were significantly higher than in Coker-312 and ZMS-12. At a lower concentration of Cr (10 μM), P_n_ values of all genotypes were higher than the control, and the highest induction (68.7%) was noted in Coker-312. At 50 and 100 μM, the P_n_ was decreased; however, the reduction was higher at 100 μM, with the highest decrease of 67.2% and 69.7% in ZMS-16 and ZMS-16w, respectively. The average data showed higher P_n_ in glandless cotton than their glanded NILs, except for 10 μM.

The G_s_ contents in untreated plants showed high genotypic variation. In ZMS-17, ZMS-16, and ZMS-12, the G_s_ contents were higher than their glandless NILs, while in glandless Coker-312w, they were higher than glanded Coker-312. Under Cr stress, a significant reduction in G_s_ was observed at a higher dose in all genotypes. At 100 μM of Cr, the highest relative decreases of 84%, 46%, and 81% were investigated in ZMS-17, ZMS-16, and ZMS-12, while they were 45%, 33%, and 63% in their glandless NILs, respectively. The results indicated that the G_s_ of the response of Coker-312 to Cr was different from other genotypes.

Like P_n_ and G_s_, the level of C_i_ decreased under higher concentrations of Cr (50 and 100 μM) in both glanded and glandless NILs. At 10 μM of Cr, ZMS-17 and Coker-312 showed a relative increase of 6.4% and 36% over their respective control, respectively. The average data showed that glanded cotton exhibited relatively higher values than their glandless NILs at all treatments. The “E” contents of untreated glanded NILs were in the range of 0.99~2.26 mmol, which had a significant variation from their glandless NILs (1.02~1.46 mmol). However, the E value in all genotypes was reduced dramatically at higher concentrations of Cr (50 and 100 μM). The reduction was more pronounced in glandless ZMS-16w and Coker 312w and glanded ZMS-12 and ZMS-17. An increase in E value was observed at 10 μM of Cr in all genotypes except Coker-312w, and the highest increase (165%) was noted in Coker-312 over its relevant control. The results revealed that at a lower dose of Cr, the plants improved their gas exchange and P_n_, while at higher Cr doses, they negatively affected the C_i_, G_s,_ and E, which ultimately decreased the plant P_n_ in all glanded and their glandless NILs. However, from average data, the P_n_ of glandless cotton was comparatively higher under 50 and 100 μM of Cr (**Table 3**).

**Table 3:** Effect of Cr on net photosynthetic rate, stomatal conductance (G_s_), intercellular CO_2_ (C_i_), and transpiration (E) of four pairs of glanded and glandless cotton NILs.

Parameters	Cr levels	NIL-1		NIL-2		NIL-3		NIL-4		Average	
		ZMS-17 (Glanded)	ZMS-17w (Glandless)	ZMS-16 (Glanded)	ZMS-16w (Glandless)	ZMS-12 (Glanded)	ZMS-12w (Glandless)	Coker-312 (Glanded)	Coker-312w (Glandless)	Glanded	Glandless
P_n_ (µmol m^−2^ s^−1^)	Control	12.8 ± 1.97^c^	12.7 ± 1.41^c^	9.17 ± 0.2^b^	9.33 ± 0.33^b^	11.68 ± 1.26^d^	16.15 ± 0.61^b^	12.28 ± 0.92^b^	10.8 ± 0.87^c^	11.5 ± 1.60^b^	12.25 ± 2.9^a^
	10 µM	20.2 ± 1.71^a^	15.2 ± 1.15^b^	11.2 ± 0.3^a^	10.9 ± 0.59^a^	14.61 ± 0.18^c^	18.94 ± 1.20^a^	20.72 ± 1.05^a^	12.9 ± 0.75^b^	16.7 ± 4.57^a^	14.50 ± 3.42^ab^
	50 µM	9.64 ± 0.9^de^	11.8 ± 1.10^cd^	6.01 ± 0.4^c^	6.52 ± 0.51^c^	8.60 ± 0.74^f^	14.24 ± 0.38^c^	9.95 ± 0.38^cd^	10.04 ± 0.30^cd^	8.55 ± 1.79^b^	10.65 ± 3.25^b^
	100 µM	8.99 ± 0.54^e^	8.30 ± 0.53^e^	3.01 ± 0.5^d^	2.82 ± 0.12^d^	8.34 ± 0.68^f^	10.32 ± 0.16^e^	8.89 ± 0.43^d^	9.40 ± 0.30^d^	7.31 ± 2.88^b^	7.72 ± 3.36^b^
G_s_ (H_2_O mol m^−2^ s^−1^)	Control	0.10 ± 0.02^a^	0.03 ± 0.00^c^	0.03 ± 0.00^a^	0.02 ± 0.00^de^	0.29 ± 0.02^a^	0.04 ± 0.00^d^	0.05 ± 0.00^b^	0.05 ± 0.00^b^	0.12 ± 0.11^a^	0.04 ± 0.01^a^
	10 µM	0.09 ± 0.02^a^	0.07 ± 0.01^b^	0.03 ± 0.00^b^	0.02 ± 0.00^c^	0.16 ± 0.01^b^	0.05 ± 0.00^e^	0.12 ± 0.00^a^	0.03 ± 0.00^c^	0.10 ± 0.04^ab^	0.05 ± 0.02^ab^
	50 µM	0.02 ± 0.00^c^	0.02 ± 0.00^c^	0.02 ± 0.00^cd^	0.01 ± 0.00^e^	0.07 ± 0.00^c^	0.02 ± 0.00^f^	0.02 ± 0.00^d^	0.01 ± 0.00^d^	0.03 ± 0.00^b^	0.02 ± 0.00^bc^
	100 µM	0.01 ± 0.00^c^	0.01 ± 0.00^c^	0.02 ± 0.00^cde^	0.01 ± 0.00^f^	0.05 ± 0.00^cd^	0.01 ± 0.00^f^	0.01 ± 0.00^d^	0.01 ± 0.00^d^	0.02 ± 0.00^b^	0.02 ± 0.00^c^
C_i_ (µmol mol^−1^)	Control	454.8 ± 4.45^b^	298.0 ± 11.1^c^	379.4 ± 5.21^a^	371.2 ± 4.40^a^	514.4 ± 14.9^a^	505.6 ± 9.77^a^	395.4 ± 0.05^c^	433.2 ± 12.2^b^	436.1 ± 61.5^a^	402.0 ± 88.5^a^
	10 µM	484.1 ± 8.00^a^	282.2 ± 14.4^d^	357.2 ± 5.74^b^	312.0 ± 5.84^c^	505.3 ± 8.41^a^	440.5 ± 9.09^b^	539.6 ± 8.8^a^	281.8 ± 20.6^d^	471.6 ± 79.6^a^	329.1 ± 75.6^ab^
	50 µM	220.0 ± 6.83^e^	141.6 ± 9.91^g^	227.1 ± 3.68^e^	247.6 ± 4.70^d^	326.8 ± 17.2^c^	309.7 ± 8.18^c^	238.8 ± 2.9^e^	215.7 ± 4.9^f^	253.2 ± 49.7^b^	228.7 ± 69.9^bc^
	100 µM	163.1 ± 6.01^f^	94.11 ± 5.92^h^	155.7 ± 3.46^f^	160.5 ± 4.55^f^	218.5 ± 13.1^d^	164.2 ± 13.6^e^	182.6 ± 17.7^g^	163.4 ± 19.8^g^	180.0 ± 28.0^b^	145.6 ± 34.4^c^
E (mmol H_2_O m^−2^ s^−1^)	Control	2.26 ± 0.05^a^	1.03 ± 0.25^c^	0.99 ± 0.10^a^	1.01 ± 0.08^a^	2.15 ± 0.18^b^	1.37 ± 0.03^c^	1.03 ± 0.05^c^	1.45 ± 0.11^b^	1.61 ± 0.69^a^	1.22 ± 0.22^a^
	10 µM	2.46 ± 0.33^a^	1.36 ± 0.23^b^	1.06 ± 0.09^a^	1.05 ± 0.04^a^	2.37 ± 0.23^a^	1.52 ± 0.07^c^	2.74 ± 0.21^a^	1.05 ± 0.12^c^	2.16 ± 0.74^a^	1.25 ± 0.23^a^
	50 µM	0.61 ± 0.09^de^	0.80 ± 0.11^cd^	0.75 ± 0.03^b^	0.44 ± 0.04^d^	0.78 ± 0.04^d^	0.63 ± 0.00^de^	0.68 ± 0.01^d^	0.55 ± 0.03^de^	0.71 ± 0.07^b^	0.61 ± 0.15^b^
	100 µM	0.49 ± 0.00^e^	0.47 ± 0.03^e^	0.63 ± 0.06^c^	0.16 ± 0.02^e^	0.58 ± 0.02^e^	0.46 ± 0.02^e^	0.50 ± 0.00^e^	0.53 ± 0.01^de^	0.55 ± 0.06^b^	0.41 ± 0.16^b^

Data are the mean of three replications (mean ± SD). Different letters show the significant differences at (*P* < 0.05) for each pair of NILs at various Cr treatments.

### 3.3 Cr accumulation in the leaves

The uptake of Cr in different cotton NILs was observed in the leaves. The results show that in the leaves of ZMS-17w and ZMS-12w, the uptake of Cr was increased with increasing concentration. However, in Coker-312 and ZMS-16, the uptake was significantly higher at 100 μM. The average data show higher translation of Cr in glandless NILs as compared to glanded NILs (**Table 4**).

**Table 4:** Cr accumulation in the leaves of four pairs of glanded and glandless cotton NILs.

Parameters	Cr level	NIL-1		NIL-2		NIL-3		NIL-4		Average	
		ZMS-17 (Glanded)	ZMS-17w (Glandless)	ZMS-16 (Glanded)	ZMS-16w (Glandless)	ZMS-12 (Glanded)	ZMS-12w (Glandless)	Coker-312 (Glanded)	Coker-312w (Glandless)	Glanded	Glandless
Cr contents in the leaves (mg g−1 DW)	CK	0.001 ± 0.00^de^	0.000 ± 0.00^e^	0.001 ± 0.00^d^	0.001 ± 0.00^d^	0.001 ± 0.00^e^	0.001 ± 0.00^e^	0.001 ± 0.00^e^	0.001 ± 0.00^e^	0.001 ± 0.00^b^	0.001 ± 0.00^b^
	10 µM	0.002 ± 0.00^de^	0.001 ± 0.00^de^	0.006 ± 0.00^cd^	0.001 ± 0.00^d^	0.002 ± 0.00^d^	0.004 ± 0.00^d^	0.002 ± 0.00^e^	0.002 ± 0.00^e^	0.003 ± 0.00^b^	0.002 ± 0.00^b^
	50 µM	0.006 ± 0.00^d^	0.034 ± 0.00^b^	0.010 ± 0.00^c^	0.027 ± 0.00^b^	0.023 ± 0.00^c^	0.017 ± 0.00^c^	0.011 ± 0.00^d^	0.015 ± 0.00^c^	0.012 ± 0.00^b^	0.024 ± 0.00^b^
	100 µM	0.018 ± 0.00^c^	0.080 ± 0.00^a^	0.051 ± 0.00^a^	0.022 ± 0.00^b^	0.034 ± 0.00^b^	0.108 ± 0.00^a^	0.056 ± 0.00^a^	0.039 ± 0.00^b^	0.040 ± 0.01^a^	0.062 ± 0.02^a^

Data are the mean of three replications (mean ± SD). Different letters show the significant differences at *P* < 0.05 for each pair of NILs at various Cr treatments.

### 3.4 Effect of Cr on the uptake of nutrient contents in the leaves

**Figure 1:**
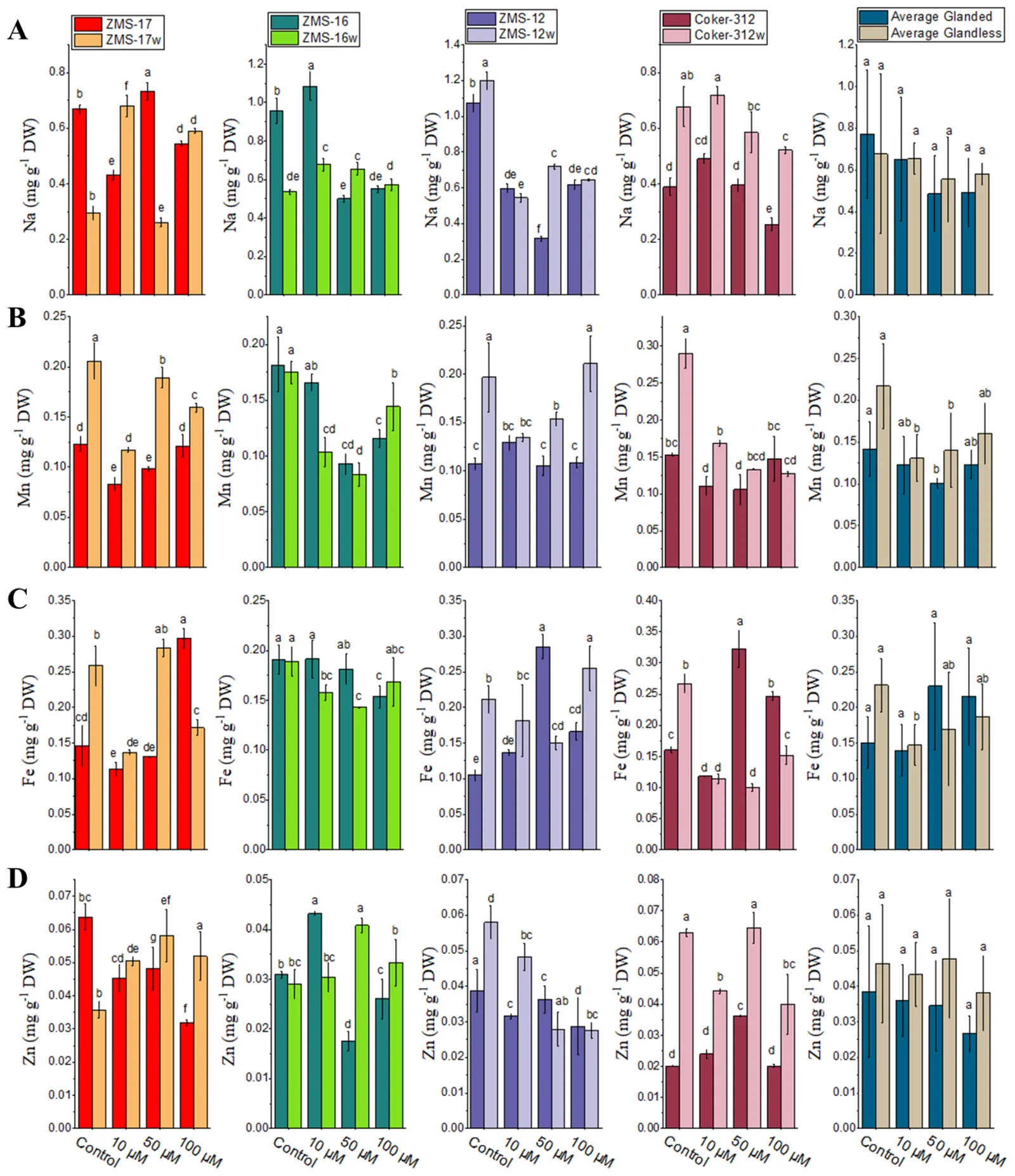
The contents of micronutrients under different Cr levels in the leaves of four pairs of glanded and glandless cotton NILs: **(A)** sodium, **(B)** manganese (Mn), **(C)** ferric (Fe), and **(D)** zinc (Zn). Different lowercase letters show significant differences between treatments (*P* < 0.05, one-way ANOVA and then Tukey’s test for multiple comparisons).

The contents of Na in the leaves of four pairs of glanded and glandless cotton NILs showed a decline when increasing the concentration of Cr as compared to the control, except for ZMS-17, which was dramatically higher than the control at 10 and 100 μM (**Figure 1A**). The Na uptake was significantly higher in ZMS-16w and ZMS-12w at 50 μM as compared to their glanded NILs. However, in Coker-312w, the Na uptake was significantly higher at all Cr concentrations. The average content of Na was higher in glandless cotton than in glanded NILs.

The Mn contents were significantly higher in glandless ZMS-17w and ZMS-12w as compared to their glanded NILs at all Cr concentrations. However, in ZMS-16w and Coker-312w, the Mn uptake was significantly higher at 100, 10, and 50 μM, respectively, than glanded NILs (**Figure 1B**). The mean average data suggest that the content of Mn was lower in the leaves of glanded cotton than in their glandless NILs (**Figure 1B**).

The contents of Fe in the leaves were significantly higher in ZMS-17w at 10 and 50 μM than their glanded NILs, whereas it was significantly higher at 100 μM in ZMS-17. In ZMS-16, its uptake was higher at 10 and 50 μM; however, at 100 μM, the absorption was significantly higher in ZMS-16w. In ZMS-12w, the Fe uptake was higher in the control and at 10 and 100 μM, whereas it was higher in ZMS-12 at 50 μM (**Figure 1C**). In Coker-312 NILs, the Fe absorption was significantly higher in glanded NILs as compared to glandless at 50 and 100 μM. The mean data indicated that glanded cotton NILs accumulated more Fe contents at 50 and 100 μM of Cr than their glandless NILs.

Zn translocation in glanded and glandless NILs was also investigated in Cr stress conditions. The results showed that the uptake of Zn in ZMS-17w and Coker-312w was higher compared to their glanded NILs at 10, 50, and 100 μM. Furthermore, it was significantly reduced by increasing Cr concentrations in ZMS-12w. The highest uptake of Zn in ZMS-12w was noted at 10 μM of Cr. However, Zn uptake was higher in ZMS-16w as compared to ZMS-16 at 50 and 100 μM, respectively (**Figure 1D**). The average data showed that glandless cotton accumulated more Zn in the leaves than their glanded NILs.

**Figure 2:**
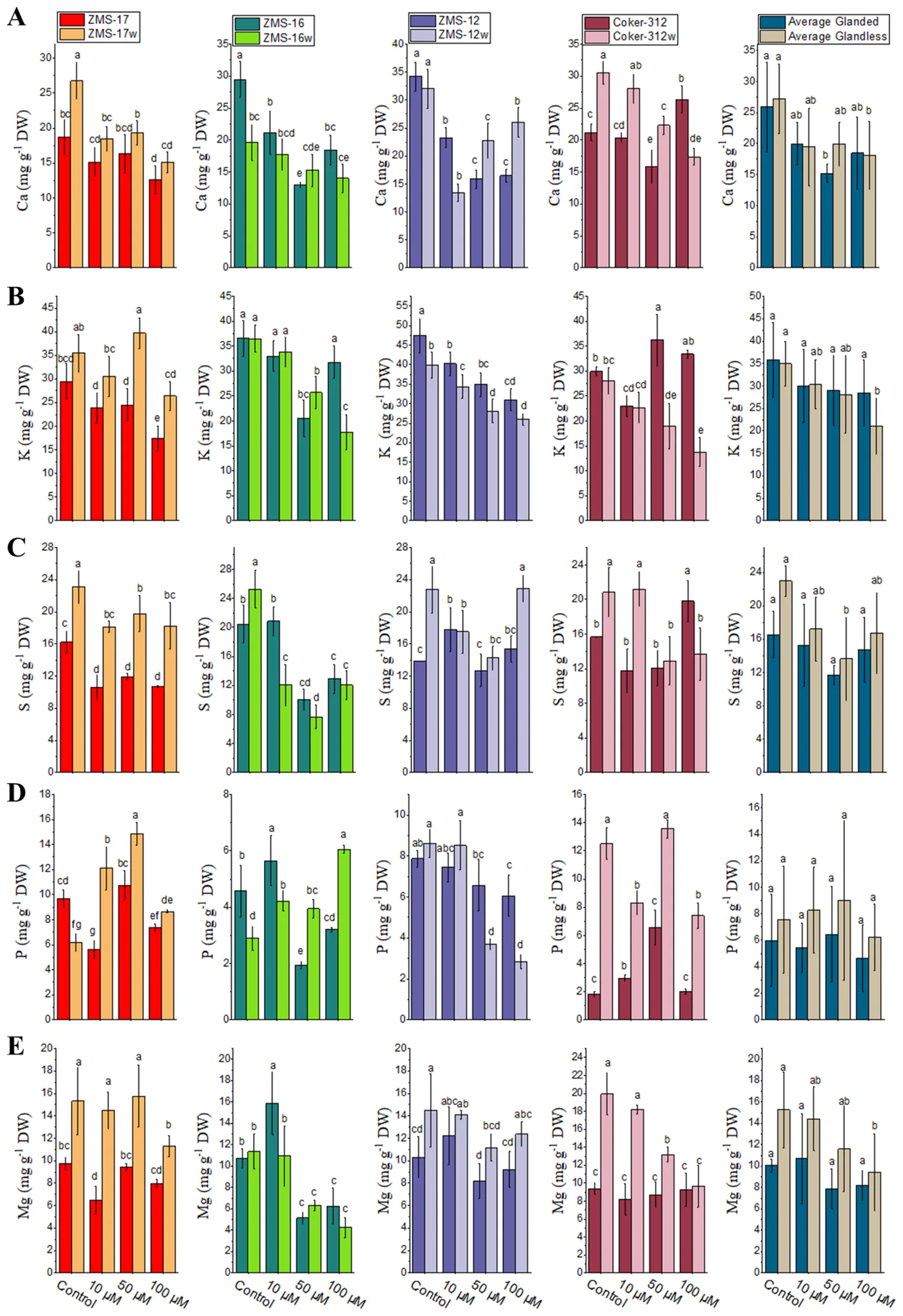
The contents of macronutrients under different Cr levels in the leaves of four pairs of glanded and glandless cotton NILs. **(A)** Calcium (Ca), **(B)** potassium (K), **(C)** sulfur (S), **(D)** phosphorus (P), and **(E)** magnesium (Mg). Different lowercase letters show significant differences between treatments (*P* < 0.05, one-way ANOVA and then Tukey’s test for multiple comparisons).

The uptake of Ca in the leaves of ZMS-17w was significantly higher at all Cr concentrations as compared to their glanded NILs (ZMS-17), while in ZMS-16w, it was higher at 50 μM as compared to ZMS-16. In ZMS-16, the uptake of Ca was significantly higher at 10 and 100 μM (**Figure 2A**). K uptake in the leaves of ZMS-17w was significantly increased with increasing Cr contents. However, K uptake was higher in the leaves of glanded NILs in ZMS-12 and Coker-312 as compared to ZMS-12w and Coker-312w (**Figure 2B**). ZMS-16w accumulated more Ca contents at 10 and 50 μM of Cr. The overall results showed that by increasing the Cr concentration, the uptake of K was reduced in the leaves of both glanded and glandless NILs as compared to the control, but the glanded NILs accumulated more K as compared to the glandless NILs.

The S contents in the leaves were significantly higher in ZMS-17w and ZMS-12w as compared to their glanded NILs at all concentrations, whereas in Coker-312w, S uptake was significantly higher at 10 μM, which was greatly reduced by increasing Cr levels from 50 to 100 μM (**Figure 2C**). However, glanded ZMS-16 NILs accumulated more S contents as compared to the glandless ones at all concentrations. The overall results showed that glandless cotton NILs accumulated more S contents than glanded NILs.

The uptake of P and Mg in the leaves of glanded and glandless NILs was investigated under Cr stress. The results show that in ZMS-17w and Coker-312w, the uptake of both P and Mg was higher than their glanded NILs (**Figures 2D, E**). In ZMS-16w, the P uptake was increased at 50 and 100 μM, whereas it was induced under 10 μM. In ZMS-12w, P uptake was higher (**Figure 2D**), while it was higher at 50 and 100 μM in glanded ZMS-12, respectively. The average data showed that the uptake of P and Mg was significantly higher in glandless NILs as compared to glanded NILs (**Figures 2D, E**).

### 3.5 Effect of Cr on photosynthetic pigments

**Figure 3:**
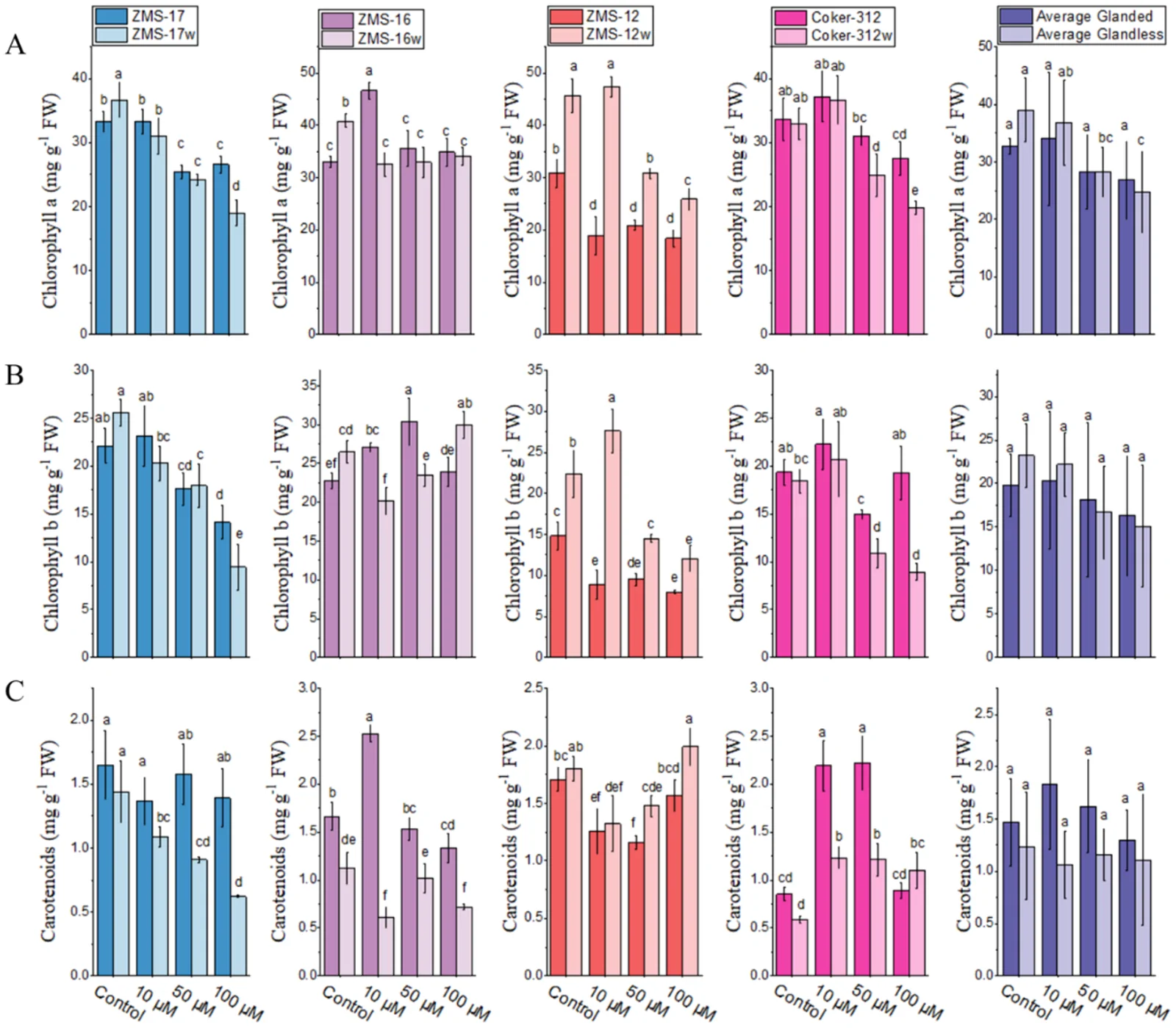
The chlorophyll contents in four pairs of glanded and glandless cotton NILs under different Cr stress levels. **(A)** Chlorophyll *a*, **(B)** chlorophyll *b*, and **(C)** carotenoids. Different lowercase letters show significant differences between treatments (*P* < 0.05, one-way ANOVA and then Tukey’s test for multiple comparisons).

Chlorophyll contents under Cr stress were examined in both glanded and glandless NILs. A significant reduction in photosynthetic pigments was observed at a higher dose of Cr in all NILs (**Figure 3**). The contents of Chl *a* were not affected by a low dose of Cr, and even an increase was observed in Coker-312, Coker-312w, and ZMS-16. However, at 50 and 100 μM of Cr, a significant reduction of Chl *a* was observed in all genotypes except ZMS-16. The highest relative decrease (48%) of Chl *a* was observed in ZMS-17w. The average data showed that Chl *a* content at 100 μM of Cr was comparatively higher in glanded cotton, while it was higher in glandless cotton at 10 μM of Cr (**Figure 3A**).

Chl *b* contents in ZMS-17w and ZMS-12 were reduced by increasing Cr concentration, while in other genotypes, a slight increase was observed at 10 μM as compared to the control. Cr significantly inhibited the Chl *b* contents at 100 µM in all genotypes except the ZMS-16 and ZMS-16w NILs. The average results indicated that Cr stress reduced Chl *b* (63%) significantly in ZMS-17w at 100 μM (**Figure 3B**).

Like Chl *a* and Chl *b*, the carotenoid contents also showed a significant genotypic variation to Cr stress. Carotenoid contents were reduced with increasing concentrations of Cr in ZMS-16/ZMS-16w, ZMS-17/ZMS-17w, and ZMS-12 NILs, except for ZMS-12w. However, in Coker-312 and Coker-312w, a significant increase was observed at all concentrations as compared to the control, and the highest induction of carotenoids at 161% was noted in Coker-312 at 50 μM of Cr (**Figure 3C**).

These results revealed that Chl *a* was inhibited more seriously by Cr stress as compared to Chl *b* and carotenoids, especially at higher doses. The contents of Chl *a* and Chl *b* were relatively higher under the control and low Cr concentration, but they were lower at a high dose in glandless cotton than in glanded NILs. Furthermore, the contents of carotenoids were high in most of the genotypes, which might contribute to the reduction of Cr toxicity and might be involved in the defense system of plants.

### 3.6 Effect of Cr on the leaf ultrastructure

**Figure 4:**
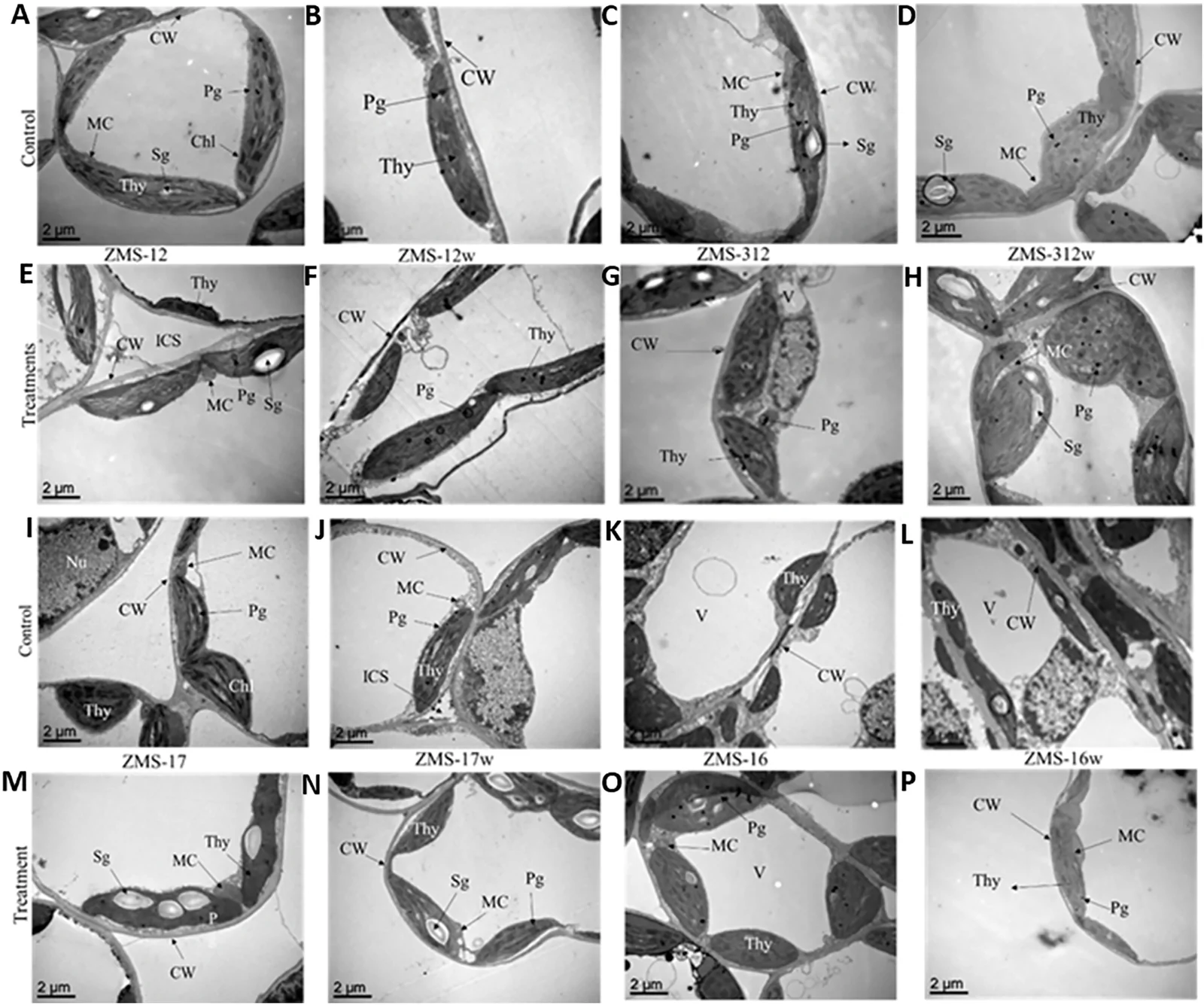
Leaves ultrastructure analysis of different glanded **(A, C, E, G, I, K, M, O)** and glandless **(B, D, F, H, J, L, N, P)** cotton NILs exposed to 50μM Cr and control. Mitochondria (MC). Chloroplast, Starch granules (Sg), Thylakoids (Thy), Plastoglobuli (Pg). Cell wall (CW.), Intercellular Spaces (ICS), Nucleus (N), Nucleolus (Nu).

Plant cell ultrastructure changes were investigated in the leaves at 50 μM because this concentration significantly affects the plant morphology as compared to the control in all glanded and glandless NILs. In ZMS-17, normal cell organelles were observed in the control as compared to the treated cells. In untreated cells, there was a prominent nucleus as well as a nucleolus inside, and well-arranged thylakoids and starch granules were observed in the chloroplast, while in Cr-treated cells, big starch granules were observed; however, the nucleus was displaced to a corner, and the cell had a thicker cell wall compared to the control. In the control plants of ZMS-17w, the cells had a well-developed chloroplasts and mitochondria and an obvious nucleus at the side of the cell. In Cr-treated plants, there were intercellular spaces between two cells, which was induced by cell shrinkage due to Cr (Cassi-lit et al., 1997). In the control cells of glanded ZMS-16, a proper oval-shaped mitochondrion was observed, while in Cr-treated plants, the mitochondria were round in shape and the cells also had intercellular spaces due to shrinkage. The same pattern was observed in glandless ZMS-16w with obvious changes in cell organs like ZMS-16. Overall, these results suggested that ZMS-17w and ZMS-16 performed better than ZMS-17 and ZMS-16w (**Figure 4**). In ZMS-12 and ZMS-12w, a normal chloroplast, a proper cell wall, and oval mitochondria were observed in the control plants, while treated plants showed a thick cell wall and had large intercellular spaces. In ZMS-16w-treated plants, a large vacuole was observed, while in untreated plants, it was normal. In untreated plants, Coker-312 and Coker-312w showed the same results, whereas the treated plants showed more starch granules and had a thick cell wall. These results revealed that ZMS-12w and Coker-312 had tolerance to Cr stress as compared to their glanded and glandless NILs (**Figure 4**).

### 3.7 Effect of Cr on lipid peroxidation, H_2_O_2_, and TSP contents

**Figure 5:**
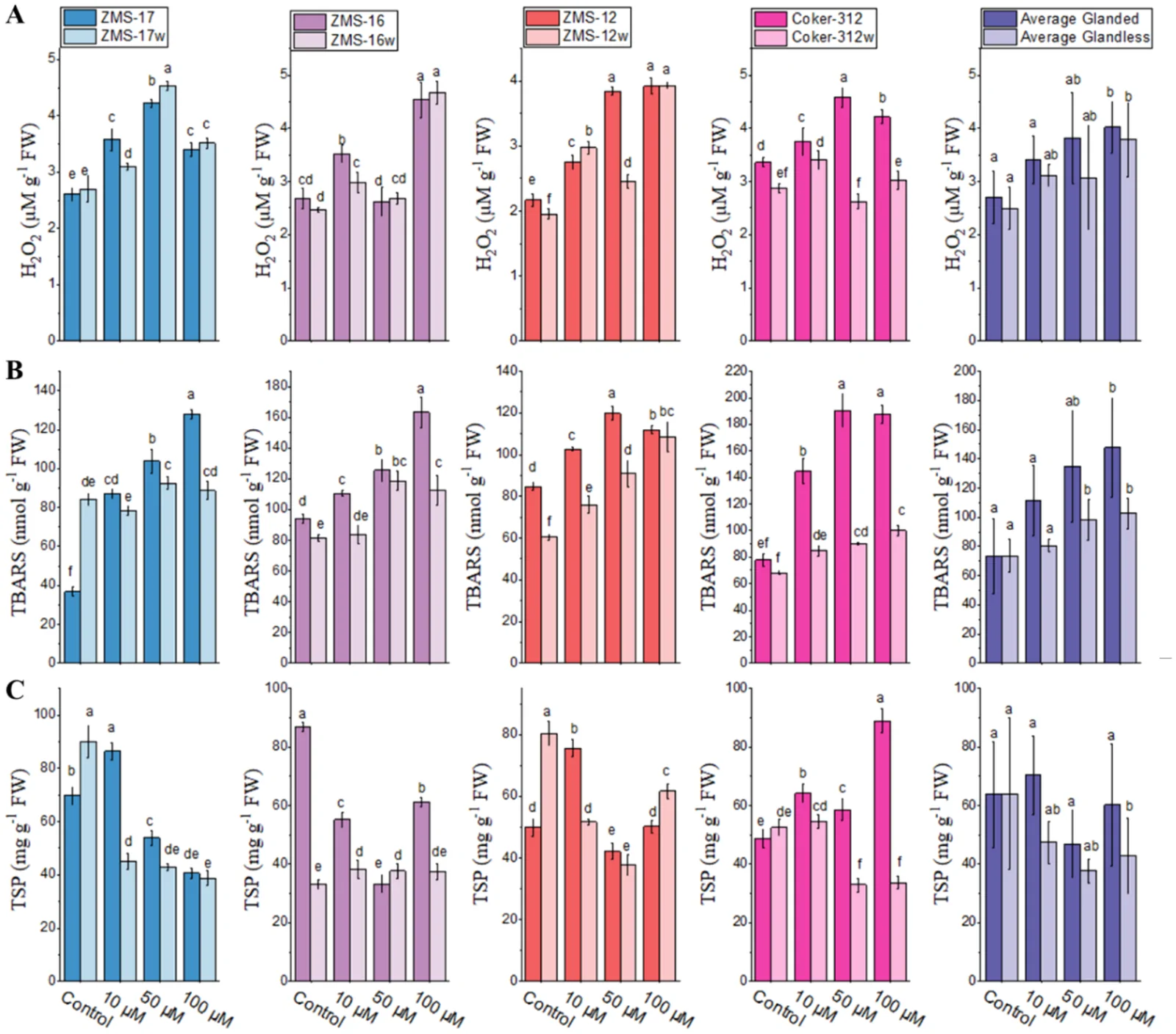
The contents of hydrogen peroxide (H_2_O_2_) **(A)**, thiobarbituric acid-reactive substances (TBARS) **(B)**, and total soluble protein (TSP) **(C)** in the leaves of glanded and glandless cotton NILs under different Cr concentrations. Different lowercase letters show significant differences between treatments (*P* < 0.05, one-way ANOVA and then Tukey’s test for multiple comparisons).

Lipid peroxidation in terms of TBARS was measured in the leaves of glanded and glandless cotton NILs (**Figure 5**). The level of H_2_O_2_ was higher under Cr stress in the leaves of all glanded and glandless NILs as compared to the control. The highest increases of 101% and 80% at 100 µM were noted in ZMS-12 and ZMS-12w, respectively. However, less increase was recorded in Coker-312w at 100 µM. The average data showed that the production of H_2_O_2_ was higher in glanded cotton than in glandless NILs at all Cr concentrations (**Figure 5A**).

TBARS contents were also significantly increased under Cr stress as compared to the control in the leaves of all genotypes except ZMS-17 at 10 µM, which showed a slight increase of 7%. The TBARS contents at 100 µM in glanded NILs were significantly increased by 249%, 140%, and 73% as compared to the control, which was higher than those of their glandless NILs (6%, 47%, and 38%). The average data showed relatively higher TBARS under Cr stress in glanded cotton than in glandless NILs at all concentrations. In the case of untreated glanded and glandless NILs, no significant changes were observed (**Figure 5B**).

TSP was also investigated in the leaves of four pairs of glanded and glandless NILs under different concentrations of Cr. The results showed that increasing the concentration of Cr significantly reduced TSP in the leaves of all glanded and glandless NILs except for Coker-312 at 100 μM, which showed an 81% increase as compared to the control. The more relative decrease of TSP contents was 61% in ZMS-16 at 50 µM. On average, the TSP contents in the leaves of glanded cotton were higher than in glandless cotton under Cr stress (**Figure 5C**).

### 3.8 Effect of Cr on antioxidant enzyme activities

**Figure 6:**
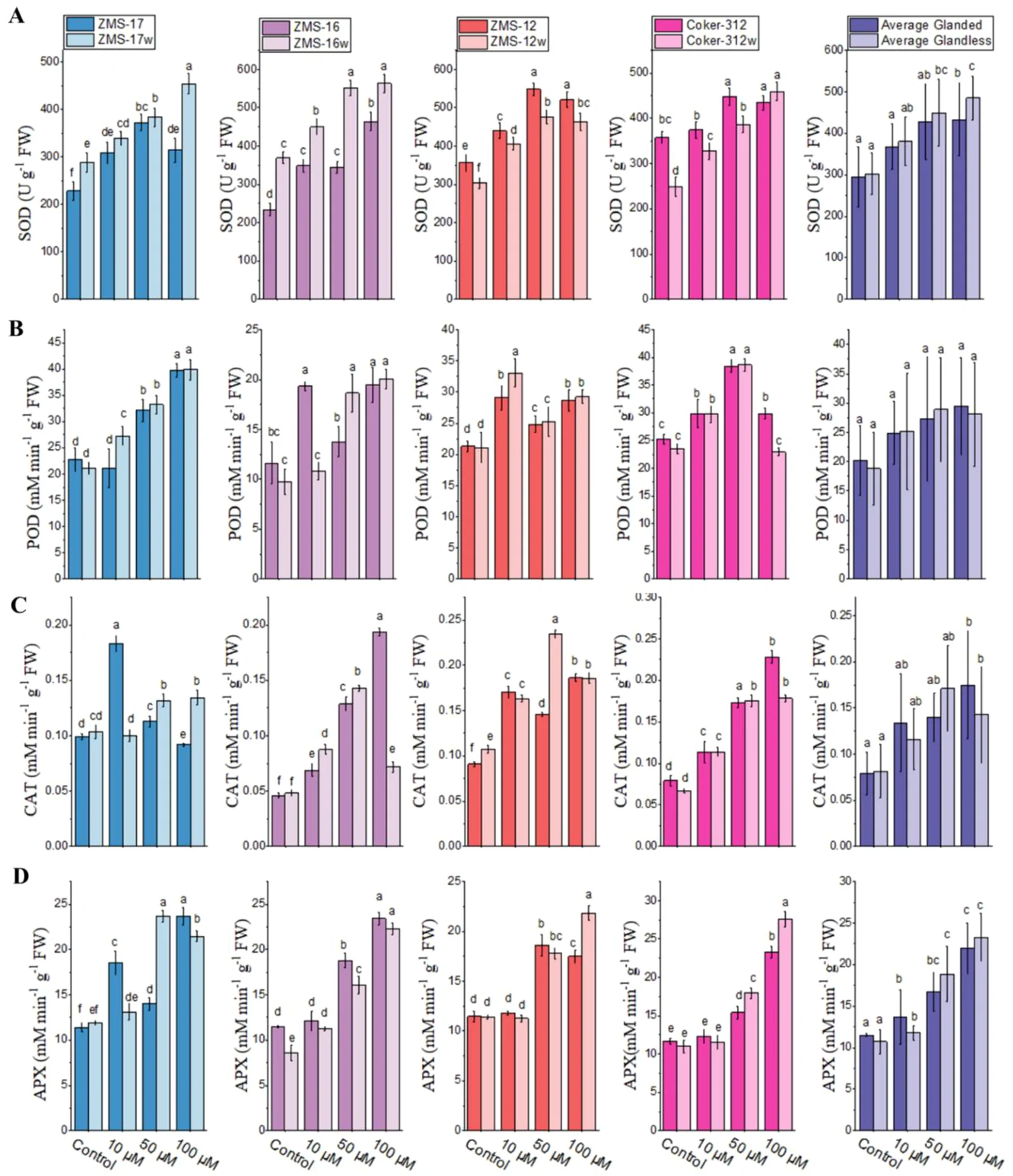
The antioxidant activities of superoxide dismutase (SOD) **(A)**, peroxidase (POD) **(B)**, catalase (CAT) **(C)**, and ascorbate peroxidase (APX) **(D)** in the leaves of four pairs of glanded and glandless cotton NILs under different levels of Cr stress. Different lowercase letters show significant differences between treatments (*P* < 0.05, one-way ANOVA and then Tukey’s test for multiple comparisons).

Antioxidant enzymes play an important role in the plant defense system by scavenging ROS, which is produced under various stresses. Cr caused a significant effect on the activities of antioxidant enzymes in the leaves of glanded and glandless cotton (**Figure 6**). SOD activity was increased with increasing Cr concentration in the leaves of all glandless NILs. The highest relative increases of 98% and 84% in ZMS-16 and Coker-312w were observed, respectively. It was relatively higher in ZMS-17w and ZMS-16w than their glanded NILs at all Cr concentrations, but it was the opposite in ZMS-12 and Coker-312 NILs. On average, the SOD level was higher in glandless cotton at all concentrations than in their glanded NILs (**Figure 6A**).

POD activity was increased with increasing Cr concentration in glandless ZMS-17w and ZMS-16w. In ZMS-12 and ZMS-12w, POD activity was increased under Cr stress, and the highest increases of 37% and 57% were observed at 10 μM in ZMS-12 and ZMS-12w, respectively. In Coker-312, the highest increases of 52% and 65% were noted at 50 μM in glanded and glandless NILs, respectively. On average, POD activity was higher in glanded cotton at 100 μM as compared to glandless NILs, but it was lower at 10 and 50 μM (**Figure 6B**).

CAT activity gradually increased with increasing concentration of Cr in ZMS-16 and Coker-312. The highest relative increase of 85% was observed in ZMS-17 at 10 μM, but it was higher in ZMS-17w at a higher concentration of Cr. CAT activity was higher at all Cr concentrations as compared to the control, and there was no difference between ZMS-12 and ZMS-12w at 10 and 100 μM. The relative increase was higher in glanded cotton at 10 and 100 μM, but it was higher in glandless NILs at 50 μM (**Figure 6C**).

The activities of APX were increased with increasing concentration of Cr in both glanded and glandless NILs of ZMS-16 and Coker-312. The relative increases (150%, 159%, and 91%) of APX at 100 μM in glandless Coker-312w, ZMS-16w, and ZMS-12w were observed as compared to the control; however, in glanded NILs, only 99%, 104%, and 52% were observed, respectively. In ZMS-17 and ZMS-17w, the highest increases of 108% and 98% at 100 and 50 μM of Cr were observed, respectively. However, on average, the activities of APX were the highest at 10 μM in glanded cotton as well as at 50 and 100 μM of Cr in glandless cotton (**Figure 6D**).

### 3.9 Impact of Cr on the expression of antioxidant genes

**Figure 7:**
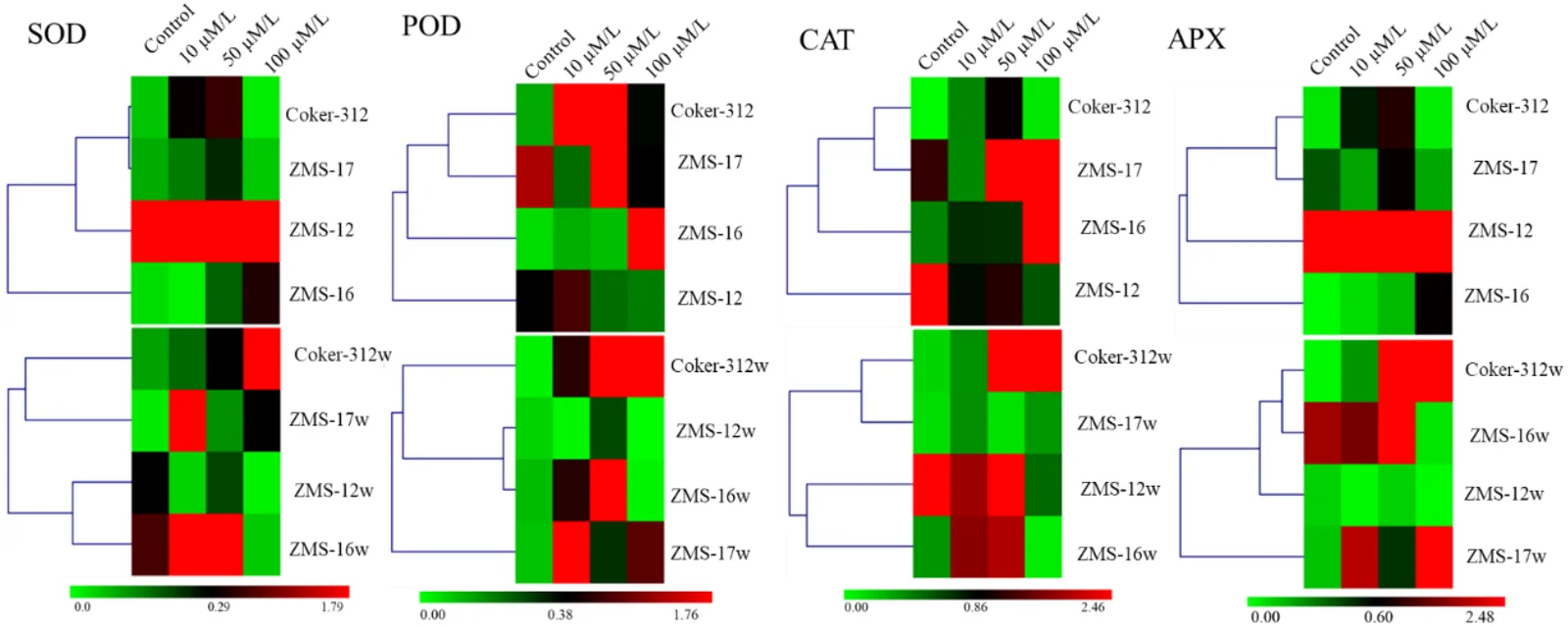
Expression level of antioxidant genes in the leaves of four pairs of glanded and glandless NILs under different levels of Cr. Superoxide dismutase (SOD), peroxidase (POD), catalase (CAT), and ascorbate peroxidase (APX). The transcription levels were normalized with actin (GhUBQ7) in each sample.

Similar to their antioxidant enzyme activities, gene expression in the leaves among glanded and glandless cotton NILs under Cr treatments for 5 days was observed (**Figure 7**). The expression level of *GhSOD* was significantly higher in the leaves of ZMS-17w and Coker 312w at 10 and 100 μM, respectively, while it was increased significantly in ZMS-16 with increasing concentration of Cr. The expression of *GhPOD* was the highest at 10 and 50 μM in ZMS-17w and ZMS-16w, respectively, while it was the highest at 50 and 100 μM of Cr in ZMS-17 and ZMS-16, respectively. Furthermore, it was significantly enhanced in Coker-312w dose dependently. The expression of *GhCAT* was high in glanded ZMS-17 and ZMS-16 at 50 and 100 μM as compared to their glandless NILs. In ZMS-12w, the expression of *GhCAT* was higher at 10 and 50 μM, while it was upregulated under all treatments of Cr in Coker 312w. The expression of *GhAPX* in Cr-treated glandless plants was significantly higher compared to glanded NILs with increasing concentration of Cr, except ZMS-12w, in which it was significantly lower under all concentrations of Cr (**Figure 7**).

## 4 Discussion

The growth and development of plants are affected by various environmental factors, i.e., nutrients and metals (López-Bucio et al., 2003). In soil and water, Cr is present at elevated levels due to mining, industrial, and other anthropogenic activities, which restrict plant nourishment and productivity. It has been stated that Cr reduced plant growth and biomass in various plants (Martínez-Trujillo et al., 2014). In the present research, we studied the effect of different concentrations of Cr on glanded and glandless cotton NILs. It was observed in the current experiment that increasing the level of Cr reduces plant growth and fresh and dry biomass of the leaves and stems (**Table 2**). These findings are in agreement with Shanker et al. (2009) and Christou et al. (2020), who stated that Cr delayed plant growth through their metabolic development. However, it mainly damages the chloroplast and cell membrane, which results in a decrease in nutrient and water uptake, thus affecting the accumulation of nitrogen and transpiration. Hence, the transport of micro- and macronutrients in plant tissues was less as compared to the control plants at all concentrations of Cr. However, the uptake of nutrients in the glanded NILs was significantly lower than glandless NILs (**Figures 1**, **2**). The absorption of Cr in glandless plants was significantly higher as compared to the glanded NILs (**Table 4**). These results are in line with the findings of Shanker et al. (2004), who stated that Cr mobilization is stopped in the vacuoles which is a natural response to natural toxicity. The higher accumulation of Cr contents might be due to sequestration in the vacuoles as a protective mechanism.

Cell walls contain proteins and polysaccharides with different functional groups that bind the metal ions and act as a first barrier that reduces the transport of metal to other parts (Zhu et al., 2013). Cr treatments affect the transport of micro- and macronutrients. However, the nutrient translocation was significantly higher in the glandless NILs compared to the glanded NILs. These results indicate that Cr competes with other nutrients to reduce its adsorption and uptake (Barbosa et al., 2007). However, plants secrete organic acids like citrate and malate that increase the solubility of metal-acting ligands (Khanna et al., 2019; Kaur et al., 2018; Kohli et al., 2018). These findings are in line with Srivastava et al. (1998), who stated that the increased translocation of Cr in the areal parts is due to the presence of citrate, aspartate, and oxalate, which convert inorganic Cr into organic, being easily available for plants.

In the present study, toxic symptoms of Cr were observed in both glanded and glandless cotton NILs through reducing the net photosynthesis rate, intercellular CO_2_, and stomatal conductance (**Table 3**). These findings are in line with Vernay et al. (2007) and Mohammed et al. (2021), who suggested that Cr targets the Calvin cycle, electron transport chain, and thylakoid, due to which the photosynthesis rate decreased. The decrease in photosynthesis rate is one of the most important toxic symptoms of heavy metal toxicity in plants (Sharma et al., 2019c; Khan et al., 2016; Shahzad et al., 2018; Sharma et al., 2019a). The reduction of chlorophyll contents was also observed under Cr stress (**Figure 3**), which suggested that the adverse toxic environment caused the lack of essential nutrients in plants and also the translocation of toxic metals to the upper parts of the plants, causing a reduction in chlorophyll contents (Khan et al., 2016; Shakoor et al., 2014; Davies et al., 2002; Qin et al., 2025). In the present study, the contents of TBARS increased with the increase of Cr concentration in all NILs, and these findings are in agreement with Ali et al. (2015); Sharma et al. (2019a), and Anjum et al. (2015b), who revealed that exposure of plants to Cr toxicity deteriorates the membrane stability through TBARS generation resulting in oxidative stress and finally an increase of H_2_O_2_. To deal with and repair the damage caused by reactive oxygen species (ROS), plants have evolved an effective and complex ROS-scavenging defense system (Daud et al., 2022; Sun et al., 2023) of enzymatic antioxidants, consisting of APX, SOD, POD, and CAT (Vajpayee et al., 2001; Dixit et al., 2002; Samantary, 2002; Zhu et al., 2023). SOD is the main enzyme that is activated initially by exposing plants to environmental stress (Shahzad et al., 2018). To scavenge ROS, SOD catalyzes superoxide and produces hydrogen peroxide (Gill and Tuteja, 2010). SOD enzymes are present in overall plant organs, therefore playing an important function in the Asada–Halliwell cycle in the chloroplast and cytosol (Bhaduri and Fulekar, 2012). While CAT, POD, and APX convert H_2_O_2_ into oxygen ions and water, they play a key role in the plant-critical environment for the scavenging of ROS (Panda and Choudhury, 2005; Mhamdi et al., 2010; Ghurbani et al., 2024; Nanehkaran et al., 2024). In the present study, we investigate that increasing the concentration of Cr significantly activates antioxidant enzymatic activities, while H_2_O_2_ contents are also increased in both NILs except ZMS-16, ZMS-16w, ZMS-12w, and Coker-312w at 50 μM (**Figure 6**). These findings are in line with Kováčik et al. (2013) and Maiti et al. (2012), suggesting that in plants, the level of H_2_O_2_ increased under Cr stress lipid peroxidation formed, and they activate antioxidant enzymatic activities. In the present study, antioxidant enzyme activities increased under Cr stress in all pairs of NILs (**Figure 6**). Generally, enhanced antioxidant enzyme activities were considered to be an adaptive strategy, which depended primarily on the type of genotype and the intensity of stress, and also worked in synchrony to reduce ROS (Shahid et al., 2017; Choudhury et al., 2013). The activities of APX were observed to be higher in cotton NILs dose-dependently, suggesting that higher production of APX may be a defense strategy to tolerate Cr toxicity. Activities of antioxidant enzymes were increased at the high dose of Cr (100 µM) (Murtaza et al., 2023) in almost all glandless NILs, showing tolerance to Cr stress, as defined by do Nascimento et al. (2018) that plants with higher antioxidant enzymatic activity were considered more tolerant to various kinds of stress. Under the low dose of Cr, activation of antioxidant enzymes was observed, hence keeping the balance between generation and elimination of ROS (Shahid et al., 2017).

Under Cr stress, TSP content was higher in glanded NILs as compared to glandless. The different increased levels of TSP showed the tolerance mechanism to Cr stress. These findings are in line with Daud et al. (2014); Karimzadeh et al. (2006). For stress regulation, ROS acts as a biological indicator (Hossain et al., 2012), activating the expression of several genes that encode proteins involved in the production, perception, and elimination of ROS in cells (Mittler, 2017; Vaahtera et al., 2014). In our findings, most of the gene expression of antioxidant enzymes was upregulated under Cr stress (**Figure 7**), which contributes to the maintenance of the activity of antioxidant compounds, eliminating ROS and strengthening the antioxidant defense system (do Nascimento et al., 2018). In addition, the immediate elimination of ROS, with the increase in oxidative stress, was found to be preferentially at its production sites, due to the activation of antioxidant substances present in this site (Hossain et al., 2012). However, the SOD and POD gene expression showed no correlation with their enzyme activities, and these findings are in line with Li et al. (2019). For the regulation of enzymes, protein modification after transcription was more important than gene expression (Fidalgo et al., 2013), because enzyme activities were regulated not only at the transcriptional level but also at posttranscriptional levels (Li et al., 2019). Differential responses of antioxidant enzymes to Cr stress indicated that antioxidant activities may play a predominant role in protection against Cr-induced oxidative stress.

## 5 Conclusion

Based on the results, it was concluded that glanded and glandless cotton might perform different mechanisms to cope with Cr toxicity. The glandless NILs showed more resistance to Cr stress as compared to glanded NILs in most aspects. Furthermore, nutrient absorption in stress conditions was significantly higher in glandless NILs as compared to glanded NILs significantly. Cr toxicity destroyed the glanded cotton NILs more severely by disturbing cell ultrastructure than the glandless NILs. We also investigated that the antioxidant activities were lower in glanded NILs as compared to glandless NILs. However, the biochemical changes were greater in glanded NILs than in their glandless NILs. The gene expression of the antioxidant-related genes was upregulated in glandless as compared to glanded NILs. To better understand the adaptive mechanism of glandless cotton under heavy metal-contaminated environments needs detailed research at the cellular and molecular levels.

## Data Availability

The contributions presented in the study are included in the article/**Supplementary Material**. Further inquiries can be directed to the corresponding author.
