# Correction: Physiological, anatomical, and molecular responses of glanded and glandless cotton to chromium exposure

**DOI:** 10.3389/fpls.2026.2010636

**Published:** 2026-10-26

**Authors:** Samrana Samrana, Abid Ali, Sayed Hussain, Hamid Ali, Uzair Muhammad, Kasim Sakran Abass

**Affiliations:** 1 College of Agriculture and Biotechnology, Zhejiang University, Hangzhou, Zhejiang, China; 2 Department of Botany, University of Swabi, Swabi, Khyber Pakhtunkhwa, Pakistan; 3 Department of Horticulture, Faculty of Chemical and Life Sciences, Abdul Wali Khan University Mardan, Mardan, Khyber Pakhtunkhwa, Pakistan; 4 Department of Bioscience, Comsats University, Islamabad, Pakistan; 5 College of Veterinary Medicine Department of Physiology, Biochemistry and Pharmacology, University of Kirkuk, Kirkuk, Iraq

**Keywords:** antioxidant activities, chromium (Cr), cotton, gene expression, gossypol, leaves ultrastructure

There was a mistake in **Figure 4** as published. Panels **I–P** duplicate the ultrastructure shown in panels **A–H**, whereas panels **4I–4P** should display different ultrastructural micrographs. The corrected **Figure 4** appears below.

**Figure 4:**
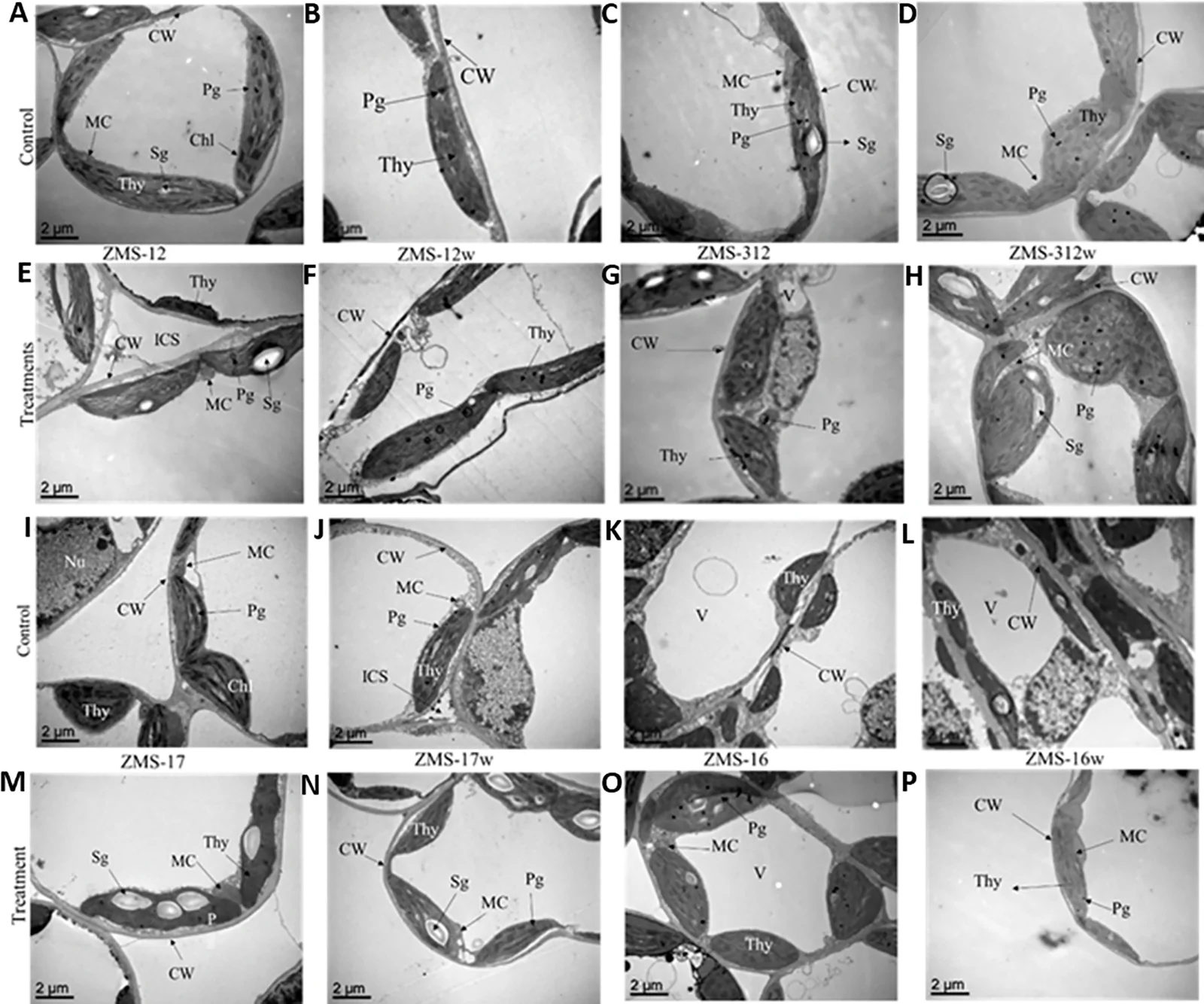
Leaves ultrastructure analysis of different glanded **(A, C, E, G, I, K, M, O)** and glandless **(B, D, F, H, J, L, N, P)** cotton NILs exposed to 50μM Cr and control. Mitochondria (MC). Chloroplast, Starch granules (Sg), Thylakoids (Thy), Plastoglobuli (Pg). Cell wall (CW.), Intercellular Spaces (ICS), Nucleus (N), Nucleolus (Nu).

The original version of this article has been updated.

