# Supplementary material for: Physiological, anatomical, and molecular responses of glanded and glandless cotton to chromium exposure
Source: Front Plant Sci. 2026 Jan 7;16:1715493. doi: 10.3389/fpls.2025.1715493 (PMC12819670; doi:10.3389/fpls.2025.1715493)
Supplement: Supplementary file 1 [file DataSheet1.pdf]

Statistix 9.0 11/17/2025,
10:51:13 AM

Completely Randomized AOV for Ci Glanded

Source	DF	SS	MS	F	P
Treatment	3	238310	79436.8	23.75	0.0000
Error	12	40144	3345.3		
Total	15	278455			

Grand Mean 335.22   CV 17.25

Completely Randomized AOV for Ci Glandless

Source	DF	SS	MS	F	P
Treatment	3	151793	50597.7	10.32	0.0012
Error	12	58846	4903.8		
Total	15	210639			

Grand Mean 276.36   CV 25.34

Completely Randomized AOV for Tr Glanded

Source	DF	SS	MS	F	P
Treatment	3	6.9603	2.32010	8.88	0.0023
Error	12	3.1353	0.26127		
Total	15	10.0956			

Grand Mean 1.2592   CV 40.59

Completely Randomized AOV for Tr Glandless

Source	DF	SS	MS	F	P
Treatment	3	2.19275	0.73092	18.46	0.0001
Error	12	0.47509	0.03959		
Total	15	2.66783			

Grand Mean 0.8716   CV 22.83

Completely Randomized AOV for conductance Glanded

Source	DF	SS	MS	F	P
Treatment	3	0.03271	0.01090	2.71	0.0914
Error	12	0.04820	0.00402		
Total	15	0.08091			

Grand Mean 0.0663   CV 95.63

Completely Randomized AOV for conductance Glandless

Source	DF	SS	MS	F	P
Treatment	3	0.00259	8.626E-04	4.61	0.0229
Error	12	0.00225	1.873E-04		
Total	15	0.00483			

Grand Mean 0.0312   CV 43.82

Completely Randomized AOV for photosynthesis Glanded

Source	DF	SS	MS	F	P
Treatment	3	208.850	69.6165	7.95	0.0035
Error	12	105.084	8.7570		
Total	15	313.933			

Grand Mean 11.011   CV 26.88

Completely Randomized AOV for photosynthesis Glandless

Source	DF	SS	MS	F	P
Treatment	3	97.727	32.5755	3.09	0.0679
Error	12	126.568	10.5473		
Total	15	224.294			

Grand Mean 11.281   CV 28.79

Completely Randomized AOV for APX glandless

Source	DF	SS	MS	F	P
Treatment	3	428.472	142.824	25.63	0.0000
Error	12	66.883	5.574		
Total	15	495.354			

Grand Mean 16.169   CV 14.60

Completely Randomized AOV for APX glanded

Source	DF	SS	MS	F	P
Treatment	3	246.472	82.1573	13.14	0.0004
Error	12	75.003	6.2502		
Total	15	321.474			

Grand Mean 15.969   CV 15.66

Completely Randomized AOV for CAT glanded

Source	DF	SS	MS	F	P
Treatment	3	0.01847	0.00616	3.33	0.0563
Error	12	0.02218	0.00185		
Total	15	0.04064			

Grand Mean 0.1319   CV 32.60

Completely Randomized AOV for CAT glandless

Source	DF	SS	MS	F	P
Treatment	3	0.01685	0.00562	3.30	0.0579
Error	12	0.02045	0.00170		
Total	15	0.03730			

Grand Mean 0.1275   CV 32.38

Completely Randomized AOV for POD glanded

Source	DF	SS	MS	F	P
Treatment	3	188.582	62.8606	1.03	0.4140
Error	12	732.358	61.0298		
Total	15	920.939			

Grand Mean 25.444   CV 30.70

Completely Randomized AOV for POD glandless

Source	DF	SS	MS	F	P
Treatment	3	248.67	82.8917	1.14	0.3736
Error	12	875.27	72.9388		
Total	15	1123.94			

Grand Mean 25.250   CV 33.82

Completely Randomized AOV for SOD glanded

Source	DF	SS	MS	F	P
Treatment	3	50690	16896.8	2.80	0.0852
Error	12	72364	6030.3		
Total	15	123055			

Grand Mean 380.76   CV 20.40

Completely Randomized AOV for SOD glandless

Source	DF	SS	MS	F	P
Treatment	3	77880	25959.9	6.90	0.0059
Error	12	45131	3761.0		
Total	15	123011			

Grand Mean 404.33   CV 15.17

Completely Randomized AOV for TBARS glanded

Source	DF	SS	MS	F	P
Treatment	3	12811.0	4270.33	4.44	0.0256
Error	12	11539.6	961.63		
Total	15	24350.6			

Grand Mean 116.73   CV 26.57

Completely Randomized AOV for TBARS glandless

Source	DF	SS	MS	F	P
Treatment	3	2286.26	762.086	6.89	0.0060
Error	12	1327.84	110.653		
Total	15	3614.09			

Grand Mean 88.669   CV 11.86

Completely Randomized AOV for TSP glanded

Source	DF	SS	MS	F	P
Treatment	3	1160.15	386.716	1.44	0.2798
Error	12	3221.96	268.497		
Total	15	4382.11			

Grand Mean 60.317   CV 27.17

Completely Randomized AOV for TSP glandless

Source	DF	SS	MS	F	P
Treatment	3	1545.80	515.267	2.28	0.1319
Error	12	2715.74	226.312		
Total	15	4261.54			

Grand Mean 48.017   CV 31.33

Completely Randomized AOV for H2O2 glanded

Source	DF	SS	MS	F	P
Treatment	3	4.02167	1.34056	3.85	0.0385
Error	12	4.17810	0.34817		
Total	15	8.19977			

Grand Mean 3.4838   CV 16.94

Completely Randomized AOV for H2O2 glandless

Source	DF	SS	MS	F	P
Treatment	3	3.33950	1.11317	2.72	0.0913
Error	12	4.91750	0.40979		
Total	15	8.25700			

Grand Mean 3.1150   CV 20.55
